# Haemocoel injection of PirA_1_B_1_ to *Galleria mellonella* larvae leads to disruption of the haemocyte immune functions

**DOI:** 10.1038/srep34996

**Published:** 2016-10-13

**Authors:** Gongqing Wu, Yunhong Yi

**Affiliations:** 1 School of Chemistry and Chemical Engineering, Guangdong Pharmaceutical University, Zhongshan, 528458, China; 2 Guangdong Cosmetics Engineering & Technology Research Centre, Zhongshan, 528458, China

## Abstract

The bacterium *Photorhabdus luminescens* produces a number of insecticidal proteins to kill its larval prey. In this study, we cloned the gene coding for a binary toxin PirA_1_B_1_ and purified the recombinant protein using affinity chromatography combined with desalination technology. Furthermore, the cytotoxicity of the recombinant protein against the haemocytes of *Galleria mellonella* larvae was investigated. We found that the protein had haemocoel insecticidal activity against *G. mellonella* with an LD50 of 131.5 ng/larva. Intrahaemocoelic injection of PirA_1_B_1_ into *G. mellonella* resulted in significant decreases in haemocyte number and phagocytic ability. In *in vitro* experiments, PirA_1_B_1_ inhibited the spreading behaviour of the haemocytes of *G. mellonella* larvae and even caused haemocyte degeneration. Fluorescence microscope analysis and visualization of haemocyte F-actin stained with phalloidin-FITC showed that the PirA_1_B_1_ toxin disrupted the organization of the haemocyte cytoskeleton. Our results demonstrated that the PirA_1_B_1_ toxin disarmed the insect cellular immune system.

*Photorhabdus luminescens*, a Gram-negative bacterium, resides as a symbiont in the gut of entomopathogenic nematodes (EPNs) of the genus *Heterorhabditis*^1^. Upon entering an insect host, EPNs release the symbiotic bacteria directly into the insect haemocoel. To infect its host and survive, bacteria must be capable of producing a wide range of proteins, including toxins^2^. To date, four primary classes of toxins are characterized in *P. luminescens*. The first class, toxin complexes (Tcs), shows both oral and injectable activity against the *Colorado potato beetle*^3^. The second class is *Photorhabdus* Virulence Cassettes (PVCs), and the injection of PVCs destroys insect haemocytes, which undergo dramatic actin cytoskeleton condensation^4^. Making caterpillars floppy (Mcf), the third class of toxins promotes apoptosis in the midgut and produces a characteristic “floppy” phenotype in the infected insect^5^. The fourth class, *Photorhabdus* insect-related toxins (PirAB), are binary toxins that exhibit injectable and oral toxicity against mosquitos and lepidopterans^6^.

To combat infection, insects rely on multiple immune responses that encompass both humoural and cellular defence reactions. Humoral reactions include the production of antimicrobial peptides (AMPs), reactive oxygen and nitrogen species, and the prophenoloxidase (proPO) activating system that regulates coagulation or melanization of haemolymph^7^,^8^,^9^. Cellular responses include the phagocytosis of small pathogens such as bacteria and fungi and the encapsulation of parasites such as parasitoids and nematodes by haemocytes^10^,^11^. The symbiotic bacteria of entomopathogenic nematodes that enter the insect haemocoel must fight with the haemocytes and AMPs. Many toxic proteins produced by symbiotic bacteria are reported to target haemocytes, which affects the host immune system by reducing vitality^12^ and by inducing apoptosis and cytolysis^13^,^14^. Moreover, in a recent study, Tc toxins inhibited the phagocytic activity of haemocytes from *Galleria mellonella*, and the biologically active components of the Tc toxins were characterized as adenosine diphosphate (ADP)-ribosyltransferases, which modify unusual amino acids and cause actin polymerization^15^.

PirAB toxin proteins, PirA_1_B_1_ and PirA_2_B_2_, are encoded at two distinct loci, *plu4093* to *plu4092* and *plu4437* to *plu4436*, in the TT01 genome, respectively, and these toxins show similarity to both δ-endotoxins from *Bacillus thuringiensis* and a developmentally regulated protein from the *Colorado potato beetle*^6^,^16^. PirA_1_B_1_ has documented oral insecticidal activity against *Plutella xylostella*, *Aedes aegypti*, *Culex pipiens* and *Gangya anopheles*, and the insecticidal activity is eliminated when *pirB*_*1*_ is knockedout^16^,^17^. Moreover, in a recent study, we demonstrated that the PirA_2_B_2_ toxin attacks haemocytes and decreases the cellular immunity of *G. mellonella* larvae following a haemocoel injection of the toxin^18^. Therefore, whether the PirA_1_B_1_ toxins also suppress the immune system of insects is a question to be investigated.

To determine the changes in the immune system of a host at the cellular level and reveal the potential role of PirA_1_B_1_ during microbial infection, we analysed the effect of the PirA_1_B_1_ toxin on the immune activity of haemocytes in *G. mellonella* larvae and investigated the most likely mechanisms. Our hope is that this study will reveal the biological role played by PirAB binary toxins in the infection process and determine their potential use in agriculture as alternatives to toxins from *Bacillus thuringiensis*.

## Materials and Methods

### Bacterial strains and insects

*Escherichia coli* DH5α was used as the host for recombinant DNA cloning, and *Escherichia coli* M15 was used as the host for expression of the PirA_1_B_1_ toxin protein. *Photorhabdus luminescens* TT01 was grown in Luria–Bertani (LB) medium at 28 °C, and the *E. coli* strains were grown in LB medium at 37 °C.

*G. mellonella* (Lepidoptera, Pyralidae) larvae were reared on an artificial diet as described in our previous study^19^. Last instar *G. mellonella* larvae with a weight of approximately 250–300 mg were used in all experiments.

### Cloning, expression and fusion protein solubility analysis

The following primers were used for amplification of *PirA*_*1*_*B*_*1*_ gene based on the genome sequence of *P. luminescens* TT01: *PirA*_*1*_*B*_*1*_-Antisense: TTTAAGCTTGTGGTGGTGGTGGTGGTGCTCAACTAATTGGTG and *PirA*_*1*_*B*_*1*_-Sense: ATAGGATCCATGCCAGTCAATCAGATTGGCTTAC (the underlined sequences denote the restriction sites). The resultant PCR products were gel purified and ligated into pMD18-T (Takara, Japan). The resultant plasmids were named pMDpirA_1_B_1_ and were transformed into *E. coli* DH5α. Three clones were sampled randomly, and the inserted DNA fragment was sequenced.

The pMDpirA_1_B_1_ was digested with *Bam*HI and *Hind*III (Takara, Japan). The inserted fragment was cloned into expression vector pQE (Qiagen, German), named pQE-pirA_1_B_1_, and then transformed into *E. coli* M15. The pQE vector without an insert fragment was selected as the control, which expressed the 6×His-tagged proteins in the prokaryotic expression system. The inserted DNA fragment was sequenced again. Cells of *E. coli* M15 harbouring pQE-pirA_1_B_1_ were grown in LB medium supplemented with ampicillin (100 mg/ml) at 37 °C to an OD_600_ nm of 0.6–0.8. Then, isopropyl-β-D-thiogalactopyranoside (IPTG) was added at a concentration of 1 mM to induce the expression of the protein. After IPTG induction for 4 h, aliquots of 1 ml of culture were sampled, and the cells were harvested by centrifugation (10,000 g, 1 min). Pellet were washed 3 times with distilled water and suspended in 0.1 ml of lysis buffer (50 mMNaH_2_PO_4_, 300 mM NaCl, 10 mM imidazole, pH 8.0), and then the cells were lysed by sonication (200–300 W, 6 × 10 s,10 s pause) and centrifuged at 10,000 g for 2 min. The supernatant was collected and 10μl aliquots were taken for SDS-PAGE.

### Purification of the fusion proteins and western blotting

Soluble proteins were directly purified by nickel affinity chromatography using a MagExtractor His-Tag NPK-700 (Toyobo, Japan), as described by the manufacturer. The resulting *E. coli* expression samples and the purified proteins were analysed by SDS-PAGE. The protein content was determined using the Bio-Rad Bradford reagent. Western blotting was then performed as described previously^18^.

### Injection and bioassays

Injections were performed directly into the insect haemocoel with a sterilized 50-μl Hamilton syringe. *G. mellonella* larvae were injected with 10 μl of PBS alone (control) or with the identical volume of PBS containing a designated amount (90, 120, 150, 180 or 240 ng/larva) of toxin protein into the haemocoel of each larva via the last left proleg. After injection, the larvae were reared on an artificial diet. Bioassays were repeated at least three times and were performed on 30 larvae for each concentration.

### Phagocytosis assays, total haemocyte counts and actin morphology

For each repeat of the experiment, two groups of *G. mellonella* larvae were injected with 10 μl of PBS containing 131.5 ng (the 50% lethal dose, LD50) of PirA_1_B_1_ or BSA per larva, and a third group were injected with PBS alone and used as a negative control. At 6 and 12 h after injection, fifteen live larvae were sampled from each group for collection of haemolymph. In each group, 40 μl of fresh haemolymph was collected from each larva by pricking the larva with an insect needle. The haemolymph from each individual was divided into two aliquots and processed as previously described for phagocytosis and total haemocyte count assays^19^.

As targets for phagocytosis, FITC-labelled *E. coli* cells were used. An aliquot of 30 μl of a haemolymph-anticoagulant solution was added to the well of a 24-well cell culture plate containing a 12-mm-diameter round glass cover slips prefilled with 300 μl of ice-cold Grace’s tissue culture medium (GIM), and incubated at 28 °C for 30 min. After several washes with GIM, the FITC-labelled *E. coli* cell suspension solution and 380 μl of GIM were added to each well and incubated for 2 h in the dark at 28 °C. Afterwards, the culture medium was removed and 300 μl of a 0.4% trypan blue solution was added to quench the nonphagocytosed bacteria. The phagocytic activity was determined by counting haemocytes with or without ingested bacterial cells under a fluorescence microscope. Five photo-frames for each microscope slide were counted to determine the average.

To count the total haemocyte number, aliquots of the haemolymph suspension were transferred to a Neubauer haemocytometer. Total haemocytes were counted using a phase contrast microscope and expressed as the number of cells per ml of haemolymph.

F-actin staining with phalloidin-FITC was performed according to previous study. Briefly, haemolymph was collected from the larvae injected with PirA_1_B_1_ (131.5 ng per larva), BSA (131.5 ng per larva) or PBS at 6 h after injection. An aliquot of 50 μl of diluted haemolymph (20 μl of haemolymph diluted in 30 μl of PBS) from each treatment group was applied to a microscope slide. The slides were placed in a moist chamber for 30 min for haemocytes to attach to the glass surface and then were fixed for 5 min in 3.7% (w/v) formaldehyde in PBS, washed three times in PBS, and then permeabilized with 0.2% Triton X-100 in PBS for 10 min at room temperature. After washing the slides with PBS, haemocyte monolayers were overlaid with phalloidin-FITC (Sigma, USA) at a concentration of 0.05 mg/ml in PBS containing1% dimethyl sulfoxide for 40 min at room temperature in a humidified chamber. Then, the slides were washed with PBS several times and overlaid with a mixture of 30% glycerol and 70% PBS (v/v) and a cover slip. Haemocytes were examined using a fluorescence microscope.

### *In vitro* toxicity experiments of PirA_1_B_1_ on haemocytes

The larvae were chilled, surface sterilized and bled into prechilled GIM, and the haemocyte density was adjusted accordingly to 1 × 10^7^ cells/ml. The haemocyte suspension was exposed to 10 μl of PBS containing 131.5 ng of PirA_1_B_1_ toxins, BSA (positive control) or PBS alone (negative control). Haemocytes were then incubated in GIM and maintained at 28 °C. After incubation for the designated time (6 and 12 h), cell morphological changes were analysed using an inverted light microscope.

### Statistical analyses

All results are expressed as the mean and standard deviation. Data were subjected to analysis of variance (ANOVA), and when the effects of ANOVA were significant, the factors that contributed to the significant differences were determined by means of least significant difference (LSD) tests using the DPS statistical software package. Significant differences were set at P < 0.05.

## Results

### Recombinant expression of PirA_1_B_1_ in *E. coli*

**Figure 1:**
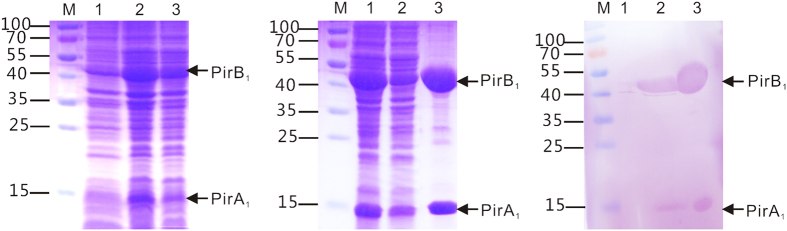
(**A**) SDS-PAGE analysis of PirA_1_B_1_ expression in *E. coli* M15 cells (pQE-pir*A*_*1*_*B*_*1*_). Lane M, markers (KDa); lane 1, uninduced pQE-pir*A*_*1*_*B*_*1*;_ lanes 2 and 3, pQE-pir*A*_*1*_*B*_*1*_induced with IPTG. (**B**) SDS-PAGE analysis of PirA_1_B_1_ expressed in *E. coli* M15 (pQE-pir*A*_*1*_*B*_*1*_) as soluble proteins followed by the purification and desalination protocols described. Lane M, markers (KDa); lanes 1 and 2, unpurified PirA_1_B_1;_ lane 3, purified and desalinated PirA_1_B_1_. (**C**) Western blot analysis of expressed protein PirA_1_B_1_ in *E. coli* M15 cells (pQE-pir*A*_*1*_*B*_*1*_). Lane M, markers (KDa); lane 1, uninduced pQE-pir*A*_*1*_*B*_*1*;_ lanes 2 and 3, pQE-pir*A*_*1*_*B*_*1*_ induced with IPTG.

The recombinant plasmid pQE-pirA_1_B_1_ was transformed and expressed in *E. coli* M15. After IPTG induction for 4 h, the whole cell lysate analysed by SDS-PAGE revealed two distinct bands with molecular weights of 45 kDa (PirA_1_) and 14 kDa (PirB_1_) (Fig. 1A), which were consistent with the predicted molecular masses. Products were not found in either the uninduced cultures or in the control (lysate of cells transfected with empty vector pQE, data not shown). PirA_1_B_1_ was successfully purified using the protocols described (Fig. 1B). The objective band of recombinant PirA_1_B_1_ was confirmed by western blot analysis with anti-His6 monoclonal antibody. For the control, no visible reaction band was detected in the group of cell lysates without IPTG induction (Fig. 1C).

### Insecticidal activity of PirA_1_B_1_

**Figure 2:**
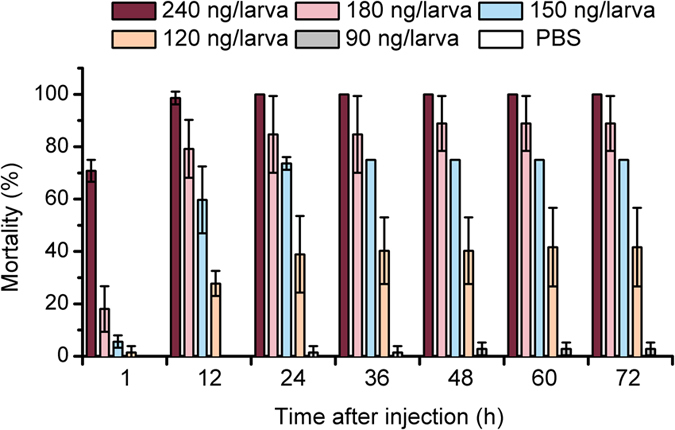
Mortality of *Galleria mellonella* larvae after injection with different doses of PirA_1_B_1_ protein.

PirA_1_B_1_ effectively killed *G. mellonella* larvae, and the mortality rate was dose-dependent based on protein concentrations. The LD50 of PirA_1_B_1_ for *G. mellonella* was 131.5 ng per larva. The larvae developed to a deep brown body colour and showed swelling symptoms after injection with PirA_1_B_1_. Larvae of *G. mellonella* injected with a high dose of PirA_1_B_1_ (240 ng per larva) died primarily during the post injection period of 1 to 12 h, with a mortality rate of 100% at 24 h after injection. No dead larvae or external symptoms were observed in the control (injected with PBS; Fig. 2).

### Injection of PirA_1_B_1_ proteins into the larval haemocoel led to decreases in numbers and phagocytic ability of haemocytes

**Figure 3:**
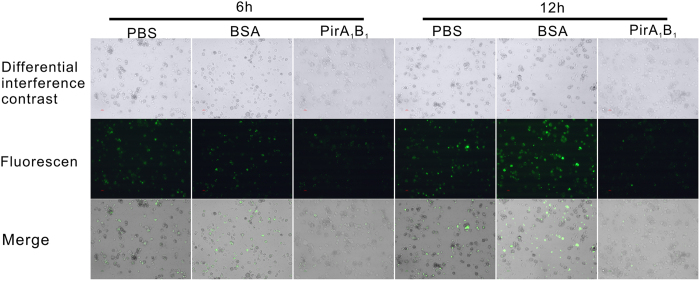
Differential interference contrast and corresponding fluorescence microscope of *Galleria mellonella* larvae haemocytes with phagocytised FITC-labelled *E. coli* cells *in vitro*. Haemocytes collected from larvae injected with PBS, BSA or PirA_1_B_1_ at 6 and 12 h after injection.

**Figure 4:**
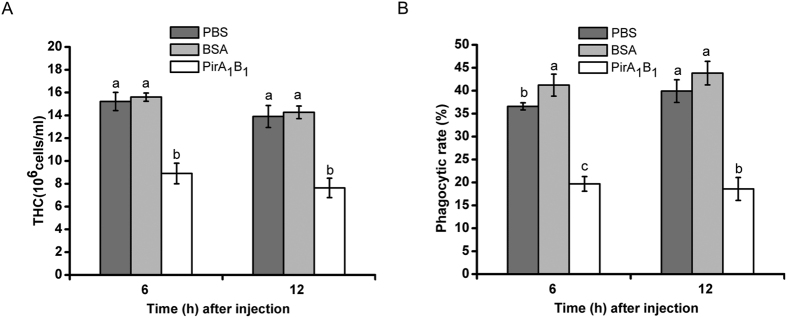
Changes in total haemocyte counts and phagocytic rates of *Galleria mellonella* larvae at 6 and 12 h after injection with PBS, BSA or PirA_1_B_1_. Values for different groups at the identical points in time followed by different letters are significantly different (P < 0.05) according to ANOVA and LSD tests.

As shown in Fig. 3, only internalized bacteria retained their fluorescence (bright green) after quenching with trypan blue. The phagocytic rate of haemocytes in the group injected with PirA_1_B_1_ protein decreased significantly compared with that in the PBS control group (P < 0.05), whereas a significant increase was detected in the BSA treatment group at 6 h after injection (P < 0.05; Figs 3 and 4B).

There was no significant difference in the total haemocyte counts (THC) between the PBS and BSA treatment groups. However, after injecting PirA_1_B_1_ protein into the *G. mellonella* haemocoel, THC decreased to 58% of the original values after 6 h and ultimately, to 54% of the initial numbers after 12 h (Fig. 4A).

### Effect of PirA_1_B_1_ protein on the cytoskeletons of the haemocytes of *G. mellonella* larvae

**Figure 5:**
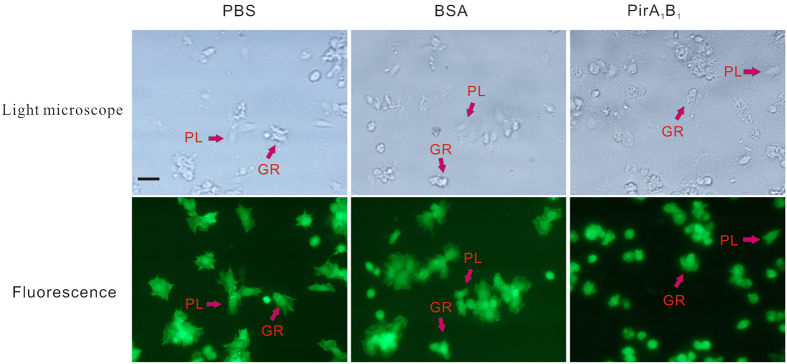
Light and corresponding fluorescence micrographs of monolayers of haemocytes from *Galleria mellonella* larvae showing localization of cytoskeletal actin stained with FITC-labelled phalloidin. Plasmatocytes (PL) and granular cells (GR) from the larvae injected with PBS or BSA showed pseudopods and an organized cytoskeleton; however, no pseudopods and spreading behaviour were observed in either plasmatocytes or granular cells from the larvae injected with PirA_1_B_1_.

Staining of haemocytes from PirA_1_B_1_-injected larvae with phalloidin-FITC showed distinct differences in actin polymerization compared with the actin-mediated assembly of the cytoskeleton in haemocytes of larvae injected with PBS and BSA. Plasmatocytes in the PBS and BSA control larvae extended their filopodia over the surface of the slide. The granular cells spread to form rounder shapes, and the actin cytoskeleton was largely organized around the periphery of these cells. However, for both plasmatocytes and granular cells in the PirA_1_B_1_-injected larvae, the cytoskeleton appeared diffuse and not organized into bundles or stress fibres (Fig. 5).

### Toxic effect of PirA_1_B_1_ protein on haemocytes of *G. mellonella* larvae *in vitro*

**Figure 6:**
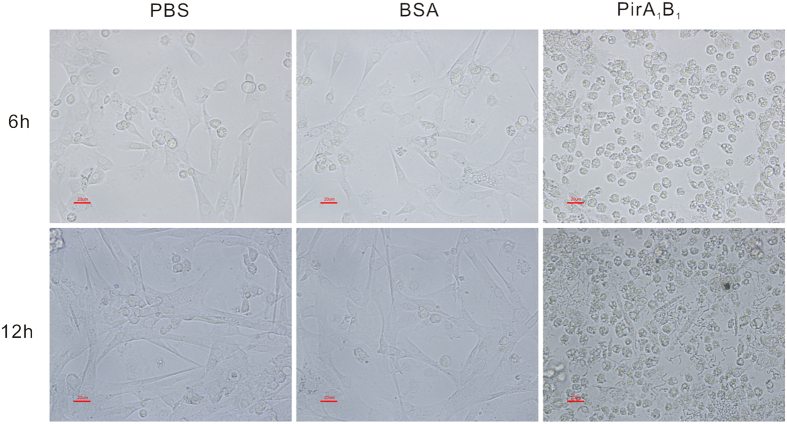
Morphology of *Galleria mellonella* larvae haemocytes treated with PBS, BSA or PirA_1_B_1_ for 6 or 12 h.

The haemocytes treated with PBS and BSA clearly spread normally and all the plasmatocytes were spindle-shaped. By contrast, most PirA_1_B_1_-treated haemocytes were small and failed to spread. After incubation for 12 h, the cytotoxic effects of PirA_1_B_1_ toxin on haemocytes were more severe, with the dead cells degenerating to form debris in the culture (Fig. 6).

## Discussion

In this study, the gene coding for the binary toxin PirA_1_B_1_ was cloned from *P. luminescens* and expressed in *E.coli* M15. The recombinant protein was purified from *E. coli* M15 using nickel affinity chromatography. The toxin protein had injection activity against *G. mellonella* with an LD50 of 131.5 ng/larva. In a previous study, the protein PirA_2_B_2_ encoded by the loci *plu4437* to *plu4436* within *P. luminescens* TT01 had haemocoel insecticidal activity against the fifth instar larvae of both *G. mellonella* and *Spodopteralitura*, with an LD50 of 4.0 and 2.8 μg/larva, respectively^20^. Compared with PirA_2_B_2_, PirA_1_B_1_ had stronger toxicity against *G. mellonella* larvae. The causes of the difference in toxicity between PirA_2_B_2_ and PirA_1_B_1_ are not clear yet. In addition to the unique biological properties of these two toxin proteins, the purification process, which can affect the structure of toxin proteins, was also an important factor worth consideration.

Haemocyte-mediated immunity is activated immediately after the insect haemocoel is penetrated by entomopathogenic nematodes. However, *P. luminescens* released from the nematode escape the insect cellular response and proliferate successfully in the haemolymph before the insect dies. A successful outcome is primarily attributed to the toxin proteins secreted by the *P. luminescens*, which fight against the haemocyte-mediated cellular immunity and protect the bacteria from the insect immune responses^21^,^22^. Recombinant *E. coli* clones carrying *plu4093*–*plu4092* (PirA_1_B_1_) of TT01 genes show oral activity against both mosquito larvae and the larvae of the moth *P. xylostella*^16^. In a recent study, PirAB-fusion protein encoded by *plu4093* and *plu4092* from *P. luminescens* TT01 also exhibited injectable insecticidal activity against *Spodoptera exigu*a larvae and had cytotoxicity against insect midgut CF-203 cells^23^. However, whether the binary toxin PirA_1_B_1_ can destroy the immune function of the insect haemocytes is unknown.

Our results showed that injection of PirA_1_B_1_ into the haemocoel of *G. mellonella* larvae significantly decreased the number of circulating haemocytes and ability for phagocytosis. The phagocytic rate and total number of haemocytes decreased to 47% and 54% of their original values at 12 h after the toxin injection, respectively. In a previous study, we also demonstrated that another toxin, PirA_2_B_2_, had similar effects on the cellular immunity of *G. mellonella* larvae^18^; however, the phagocytic rate decreased to 71% of the PBS control at 12 h after injection and did not change afterwards, which indicated higher toxicity of the toxin PirA_1_B_1_ against *G. mellonella* larvae. Haemocytes play important roles in phagocytosis and capsule formation^11^,^24^, and with more haemocytes, insect have greater ability to clear invading pathogens^25^. Therefore, with the decrease in haemocytes, we believe that the immune strength and immune system coordination were weakened directly. The mechanism by which PirA_1_B_1_ causes this reduction in circulating haemocyte number is currently unknown. One possibility is the reduction in haemocyte number due to death and disintegration of haemocytes and/or reduced proliferation of haemocytes^26^. We performed *in vitro* experiments and directly treated haemocytes with PirA_1_B_1_, and the results showed that the toxin disrupted the spreading behaviour of haemocytes at 6 h after treatment. Furthermore, after incubation for another 12 h, the PirA_1_B_1_ toxin caused the haemocytes to lyse, which degenerated to form debris in the culture. We also observed that treatment with the PirA_1_B_1_ toxin caused a reduction in haemocyte pseudopod formation. Therefore, based on our results, the binary toxin PirA_1_B_1_ was cytotoxic against the haemocytes of *G. mellonella* larvae. These results were consistent with the *in vivo* experiments in which haemocoel injection of PirA_1_B_1_ caused significant reductions in number of circulating haemocytes in *G. mellonella* larvae.

To investigate the mechanisms responsible for the inhibition of phagocytosis by haemocytes after PirA_1_B_1_ treatment, we performed experiments to determine whether the toxin altered the haemocyte cytoskeleton. Staining the haemocytes with FITC-labelled phalloidin revealed major differences in the cytoskeletal architecture of cells following injection of the toxin protein compared with those in BSA or PBS control larvae. These assays indicated that PirA_1_B_1_ adversely affected the cytoskeleton of *G. mellonella* haemocytes, causing it to become diffuse and disorganized, and this result also correlated with the reduction in formation of pseudopods by the cells. Therefore, we concluded that the toxin PirA_1_B_1_ disrupted the cytoskeletons of haemocytes, which led to abnormal haemocyte spreading behaviour and pseudopod formation. Because haemocyte spreading behaviour and the ability to extend pseudopods are essential for phagocytosis^27^, with their disruption, phagocytosis was suppressed. Thus, PirA_1_B_1_ inhibited phagocytosis by affecting the cytoskeleton of the immunocytes. Numerous bacterial toxins reorganize the actin cytoskeleton of target cells. Moreover, *Photorhabdus* W14 bacterial supernatants cause marked changes in the actin cytoskeleton of specific haemocyte types^22^. Additionally, the injection of recombinant *E. coli* expressing *Photorhabdus* virulence cassettes (PVC) containing cosmids from *Photorhabdus* destroys insect haemocytes, which undergo dramatic actin cytoskeleton condensation^4^.

In conclusion, based on our preliminary results, following the injection of PirA_1_B_1_ toxin into the haemocoel of *G. mellonella* larvae, the host haemocyte number decreased and the ability of haemocyte phagocytosis was inhibited by the disruption of the cytoskeleton. Therefore, the toxin PirA_1_B_1_ disarmed the insect cellular immune system. As a ubiquitous protein in entomopathogenic nematode symbiotic bacteria, uncovering the cellular immune responses of a host insect to the injection of PirA_1_B_1_ helped to understand the interaction between nematode-symbiotic bacteria and their hosts.

## Additional Information

**How to cite this article**: Wu, G. and Yi, Y. Haemocoel injection of PirA_1_B_1_ to *Galleria mellonella* larvae leads to disruption of the haemocyte immune functions. *Sci. Rep.* **6**, 34996; doi: 10.1038/srep34996 (2016).
